# Five *OsS40* Family Members Are Identified as Senescence-Related Genes in Rice by Reverse Genetics Approach

**DOI:** 10.3389/fpls.2021.701529

**Published:** 2021-09-03

**Authors:** Habiba, Jiaxuan Xu, Ahmed G. Gad, Yuling Luo, Chunlan Fan, Junaite Bin Gias Uddin, Noor ul Ain, Chengxin Huang, Yu Zhang, Ying Miao, Xiangzi Zheng

**Affiliations:** Fujian Provincial Key Laboratory of Plant Functional Biology, College of Life Sciences, Fujian Agriculture & Forestry University, Fuzhou, China

**Keywords:** *OsS40* gene family, leaf senescence, rice, CRISPR/Cas9 edited mutants, gene expression network

## Abstract

A total of 16 *OsS40* genes of *Oryza sativa* were identified in our previous work, but their functions remain unclear. In this study, 13 *OsS40* members were knocked out using the CRISPR/cas9 gene-editing technology. After screening phenotype characterization of CRISPR/Cas9 mutants compared to WT, five *oss40s* mutants exhibited a stay-green phenotype at 30 days after heading. Moreover, increased grain size and grain weight occurred in the *oss40-1, oss40-12*, and *oss40-14* lines, while declined grain weight appeared in the *oss40-7* and *oss40-13* mutants. The transcript levels of several senescence-associated genes (SAGs), chlorophyll degradation-related genes (CDGs), as well as WRKY members were differentially decreased in the five stay-green *oss40s* mutants compared to WT. Five *oss40* mutants also exhibited a stay-green phenotype when the detached leaves were incubated under darkness for 4 days. *OsSWEET4* and *OsSWEET1b* were significantly upregulated, while *OsSWEET1a* and *OsSWEET13* were significantly downregulated in both *oss40-7* and *oss40-14* compared to WT. Furthermore, these five *OsS40* displayed strong transcriptional activation activity and were located in the nucleus. Most of the *OsS40* genes were downregulated in the *oss40-1, oss40-7*, and *oss40-12* mutants, but upregulated in the *oss40-13* and *oss40-14* mutants, indicating coordinated regulation among *OsS40* members. These results suggest that *OsS40-1, OsS40-7, OsS40-12, OsS40-13*, and *OsS40-14* are senescence-associated genes, involved in the senescence and carbon allocation network by modulating other *OsS40* members, *SWEET* member genes, and senescence-related gene expression.

## Introduction

Senescence is a natural phenomenon that is clearly marked by leaf color changes due to the reduction in leaf functionality. It is a highly coordinated developmental process during which functional compounds from old leaves are degraded to release valuable nutrients that are redistributed into developing tissues *via* the differential expression of senescence-associated genes (SAGs) (Buchanan-Wollaston et al., 2005; Pyung et al., 2007). The onset of leaf senescence begins with major physiological alterations, including an increase in the breakdown of chlorophyll and a switch from carbon assimilation to catabolism of energy resources, such as proteins, lipids, and nucleic acids (Pyung et al., 2007; Liu et al., 2008). Delayed leaf senescence is usually accompanied by photosynthetically active and a prolonged flowering period, enabling plants to produce more seeds and accumulate more biomass (Guo and Gan, 2014). Therefore, understanding the underlying mechanism of leaf senescence for rice breeding is important.

In the last few decades, a series of SAGs have been isolated and characterized in rice, including transcription factors, receptors and signaling components of hormones or stress responses, and metabolic regulators (Yamada et al., 2014). Transcription factors (TFs) involved in the regulation of SAGs have been characterized mainly in *Arabidopsis thaliana*. These transcription factors are mostly members of the WRKY, NAC, C2H2 zinc finger, MYB, and AP2-EREBP families (Guo et al., 2004; Buchanan-Wollaston et al., 2005; Balazadeh et al., 2008; Breeze et al., 2011; Gregersen et al., 2013; Christiansen and Gregersen, 2014). Senescence-related transcription factors have also been discovered in rice, primarily among members of the WRKY, NAC, and MYB families (Luoni et al., 2019). For example, *OsNAP*, a member of the NAC family, is induced by ABA and directly upregulates chlorophyll degradation genes, including *SGR, NYC1, NYC3*, and *RCCR*1, leading to early leaf senescence (Liang et al., 2014). OsWRKY5 promotes leaf senescence under natural and dark-induced senescence conditions (Kim et al., 2019), and OsWRKY42 enhances leaf senescence by repressing the expression of OsMT1d to induce reactive oxygen species in rice (Han et al., 2017). Natural aging and dark-induced SAGs *Osh36* and *Osl85* encode aminotransferase and isocitrate lyase, which are involved in amino acid and fatty acid metabolism, respectively (Lee et al., 2001). Analysis of SAGs expression profiles indicates there is a complex regulatory network for leaf senescence processes (Shimoda et al., 2016; Yang et al., 2016; Zhao et al., 2016; Deng et al., 2017; Hong et al., 2018; Ke et al., 2019) and that some key regulators play an active role in the regulation of senescence. So far, most of the progress in understanding senescence has come from the model dicot *A. thaliana*, while limited information of SAGs from rice is known.

One of the most effective approaches to understanding leaf senescence mechanisms is the isolation and analysis of stay-green (also termed non-yellowing) mutants, which show a delayed leaf senescence phenotype (Jiang et al., 2007; Thomas and Ougham, 2014; Zhao et al., 2019). To date, in model and crop plant species, a small number of stay-green mutants have been identified (Hörtensteiner, 2009; Kusaba et al., 2013; Zhao et al., 2019). Another approach is reverse genetics screening, and several reverse genetics studies have revealed that chlorophyll degradation genes (CDGs) contribute to sequential chlorophyll degradation reactions during leaf senescence. *STAY-GREEN* (*SGR*), a typical CDG, regulates chlorophyll degradation metabolic pathways by inducing disassembly of photosystem II (LHCPII) and also functions as magnesium (Mg) dechelatase catalyzing the extraction of Mg from chlorophyll a during chlorophyll degradation (Hörtensteiner, 2009; Shimoda et al., 2016; Xu et al., 2019). *NON-YELLOW COLORING 1* (*NYC1*) encodes the chlorophyll b oxidation-reduction enzyme. In the *nyc1* mutant, chlorophyll b cannot be degraded because of the abnormal binding of light-harvesting chlorophyll (Kusaba et al., 2007). Overexpressing OsNYC1 in rice (*Oryza sativa*) can induce the degradation of chlorophyll (Sato et al., 2009). Pheophorbide a oxygenase (*OsPAO*) knockdown in rice leads to accumulation of pheide a and prolongs the greenness of the leaf during dark incubation (Tang et al., 2011).

A set of regulatory genes induced explicitly during leaf senescence belongs to the *S40* gene family, which encodes the plant-specific domain of unknown function 584 (DUF584)-harboring proteins in *Arabidopsis thaliana* (L.) Heynh., *Hordeum vulgare* L., and *Oryza sativa* L. ssp. *japonica* (Kleber-Janke and Krupinska, 1997; Krupinska et al., 2002; Fischer-Kilbienski et al., 2010; Zheng et al., 2019). Barley HvS40 is highly induced in various situations causing senescence, ranging from (a) biotic-induced senescence to age-dependent senescence of barley leaves (Becker and Apel, 1993; Humbeck et al., 1996; Kleber-Janke and Krupinska, 1997; Krupinska et al., 2002; Gregersen et al., 2008). In *Arabidopsis*, the nuclear-localized AtS40-3 seems to be a key regulator of leaf senescence by repressing the expression of the central senescence regulatory gene *WRKY53* (Miao et al., 2004) and two SAG markers, *SAG12* and *SEN1*. The loss-of-function mutant of AtS40-3 leads to a stay-green phenotype under both natural and dark-induced leaf senescence conditions (Fischer-Kilbienski et al., 2010). In rice, expression profiles of all 16 *OsS40* genes during the natural senescence of flag leaf and under various senescence-promoting stress treatments showed a differential response level. Among them, *OsS40–1, OsS40–2, OsS40–12*, and *OsS40–14* were highly stress-responsive, which suggests a potential regulatory function in rice senescence cross-talk between abiotic, biotic, and developmental senescence (Zheng et al., 2019). Although this information counts as a valuable foundation to understand the regulatory function of *OsS40* genes in leaf senescence, their biological significance in the natural senescence of rice remains unclear.

In this study, by using CRISPR/Cas9 gene-editing technology, 13 *OsS40* gene family members (*oss40-1, oss40-2, oos40-4, oos40-5, oos40-6, oss40-7, oss40-8, oss40-9, oss40-11, oss40-12, oss40-13, oss40-14*, and *oss40-15*) were mutated, and their edited mutants were used for systematic characterization of the *OsS40*s during natural senescence of rice leaves. Five *OsS40* member mutants showed a delayed flag leaf senescence (stay-green) phenotype and dark-induced senescence of detached leaves and displayed a distinct effect on grain weight. These five senescence-related members were further characterized with regard to their transcriptional activity and subcellular localization. Meanwhile, genetic analysis revealed complex relationships among *OsS40* members and a subgroup of *OsS40* genes implicated in natural senescence and dark-induced senescence of rice leaves. These results provide important clues to the mechanism underlying rice leaf senescence.

## Materials and Methods

### Plant Materials and Growth Conditions

The *oss40* mutants and their parental WT Nipponbare (*Oryza sativa* L. ssp. *japonica*) and *Oryza sativa* cv. CO39 for rice protoplast were grown under 16 h light at 30°C/8 h night at 22°C greenhouse conditions. Seeds were germinated in wetted filter paper at 37°C for 2 days in darkness and then grown in a growth chamber condition after transplanting in small plastic pots filled with an equal amount of vermiculite and compost. For all the experiments, rice seedlings were grown in small pots of similar sizes (five plants per pot). For expression profiling analysis, same-germination-stage, 1-month-old plant leaf samples were collected from the WT and knockout mutants. Thirty-day-old seedlings were then transplanted, one plant per big plastic pot containing field soil. Fertilizer was applied (nitrogen–phosphorus–potassium) two times in the growth period during transplantation in a big pot and after the heading stage. After the heading initiation, the flag leaves were tagged to enable identification of the same stage leaf to compare visible phenotype and chlorophyll measurement between mutants and the wild type.

### Identification of *Oss40* Mutants by CRISPR/Cas9 Gene Editing

The *OsS40* genes CRISPR-edited plants were generated with the aid of Hangzhou Biogle Co., Ltd. (Hangzhou, China), following a method described previously by Wang et al. (2015). The target gRNA sequences of *OsS40-1*–*OsS40-16* are listed in Table 1. To identify the mutants, genomic DNA was isolated using Edward's buffer from mutant leaves (Chen and Kuo, 1993). PCR amplification was performed using specific primers to amplify the genomic area surrounding the CRISPR target sites (Supplementary Table 1).

**Table 1:**
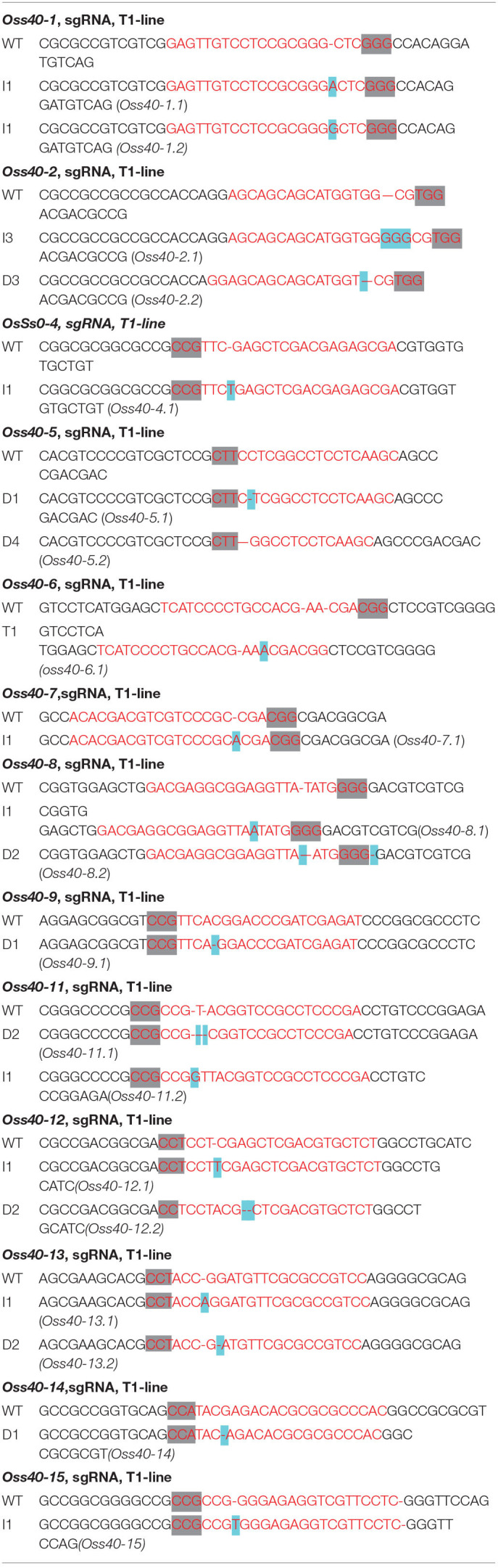
The targeted sequence region of *OsS40*s CRISPR/Cas9-edited mutants.

*Red color indicates cas9 leading sequence; blue color indicates edited nucleotide. I1, inserted line; D, deleted; T1, first progeny. Grey box indicates PAM motif (NGG)*.

### Gene Expression Analysis by Quantitative RT-PCR

For detection of tissue-specific gene expression, young leaves, mature leaves, roots, stems, and seeds of WT Nipponbare (NIP) were used for RNA extraction. Young leaves, stems, and roots were collected from 2-week-old seedlings under greenhouse conditions. Mature leaves were obtained at the floral induction stage. For detection of senescence-associated gene expression in the *oss40* mutant lines, total RNA was extracted by TRIzol, using primary leaves of the 1-month-old *oss40* mutant line, referring to the instructions of the manufacturer (Invitrogen). The first-strand cDNA was obtained from 1 μg of total RNA, using the Synthesis Kit (Thermo Fisher Scientific), eliminating the contaminant genomic DNA. qRT-PCR was performed to analyze the expression of genes in the CFX96 machine (Bio-Rad Company, Hercules, CA, USA) in a whole volume of 15 μl, using SYBR Green Master (ROX) (Newbio Industry, China) as per the instructions of the manufacturer. The primer sequences used for expression analysis of the *OsS40s* were as described in Zheng et al. (2019). The endogenous *OsACTIN* gene (*LOC_Os03g50885*) was used as the reference gene. Relative expression levels of target genes were calculated using the 2^−ΔΔCt^ method as mentioned (Schmittgen and Livak, 2008). In this experiment three biological replicates were tested, and each biological replicate contains leaves from three independent plants.

### Co-localization of the Fusion Proteins and Confocal Microscopy

One of the selected senescence-related WRKY gene, *OsWRKY44* (*LOC_Os03g21710*) (Li et al., 2021), was cloned into the entry vector pDONR201, followed by recombination into the expression vectors p2GWF7 (C-terminal GFP fusion) and p2GWR7 (C-terminal RFP fusion), using the Gateway® cloning technology as previously described by Zheng et al. (2019). The expression vectors of *OsS40*s-GFP were previously described by Zheng et al. (2019). For the co-localization assay, *OsS40*s-GFP and OsWRKY79-RFP were co-transformed into rice protoplasts according to the description (Zheng et al., 2019) and incubated overnight at room temperature in darkness. All microscopic observations were done using a Leica TCS SP8 confocal laser scanning microscope as previously described (Zheng et al., 2019). Images were processed with Leica TCS SP8 confocal software. Figures were organized with Adobe Photoshop and Adobe Illustrator.

### Transcriptional Activity Assay in Rice Protoplasts

The transcriptional activation assay was performed using a Dual-Luciferase Reporter Assay System (Ohta et al., 2001) in rice protoplast. A pUC19 vector containing a firefly LUC gene under five GAL4-binding elements fused to the promoter was used as a reporter plasmid. A pTRL vector, including a Renilla LUC gene, was used as an internal control (Ohta et al., 2001). Coding sequences of *S40* genes were amplified using the specific primers (Supplementary Table 1), and the PCR products were digested by ApaI/Kpn I and cloned into a pRT-BD vector to generate *35S-BD-OsS40s* (effector plasmids). The effectors, reporter, and internal control plasmid were co-transfected into protoplasts (about 2.5 × 10^6^ cells/ml) isolated from rice primary leaves. After culturing for 12–16 h, luciferase assays were performed according to Fraiture et al. (2014). Luciferin was added to 600 μl of transfected protoplast solution at a final concentration of 200 μM for the luciferase assay. Luciferase assay was measured using a microplate reader (VarioSkan Flash-Thermo scientific), and each experiment had three biological replicates with at least three-time technique repetition.

### Measurements of Chlorophyll Fluorescence and Concentration

Chlorophyll fluorescence of living leaves was measured using Pocket PEA Chlorophyll Fluorimeter (Hansatech Instruments, Norfolk, UK) as described previously by Zheng et al. (2019). Chlorophyll concentration in living leaves was determined with a portable chlorophyll content meter (CCM 200 plus, Opti-Sciences, Hudson, NH) as described previously by Zheng et al. (2019), which determined the relative content of chlorophyll by measuring the difference in optical density at two wavelengths (650 and 940 nm) and automatically calculated the value. Three to five leaves of each plant were used in these chlorophyll assays. Each leaf, top-to-middle, part was measured, and the average value was calculated. The chlorophyll content of the detached leaves was measured based on the method described previously (Lichtenthaler and Wellburn, 1983; Fatima et al., 2021). The absorbance of extracted pigments was measured at 470, 649, and 665 nm by spectrophotometer (L3, INESA, China), and the total chlorophyll concentration was calculated following the equations mentioned (Lichtenthaler and Wellburn, 1983).

### Statistical Analysis

The data in all figures were determined by at least three biological replicates. One-way ANOVA was performed to understand the differences in levels of gene expression among distinct rice tissues. The Student's *t*-test was used for mean comparisons. All these were carried out using the GraphPad Prism software version 8 (GraphPad Software, San Diego, CA, USA).

## Results

### Tissue-Specific Expression Profiles of **OsS40** Genes in Rice

**Figure 1:**
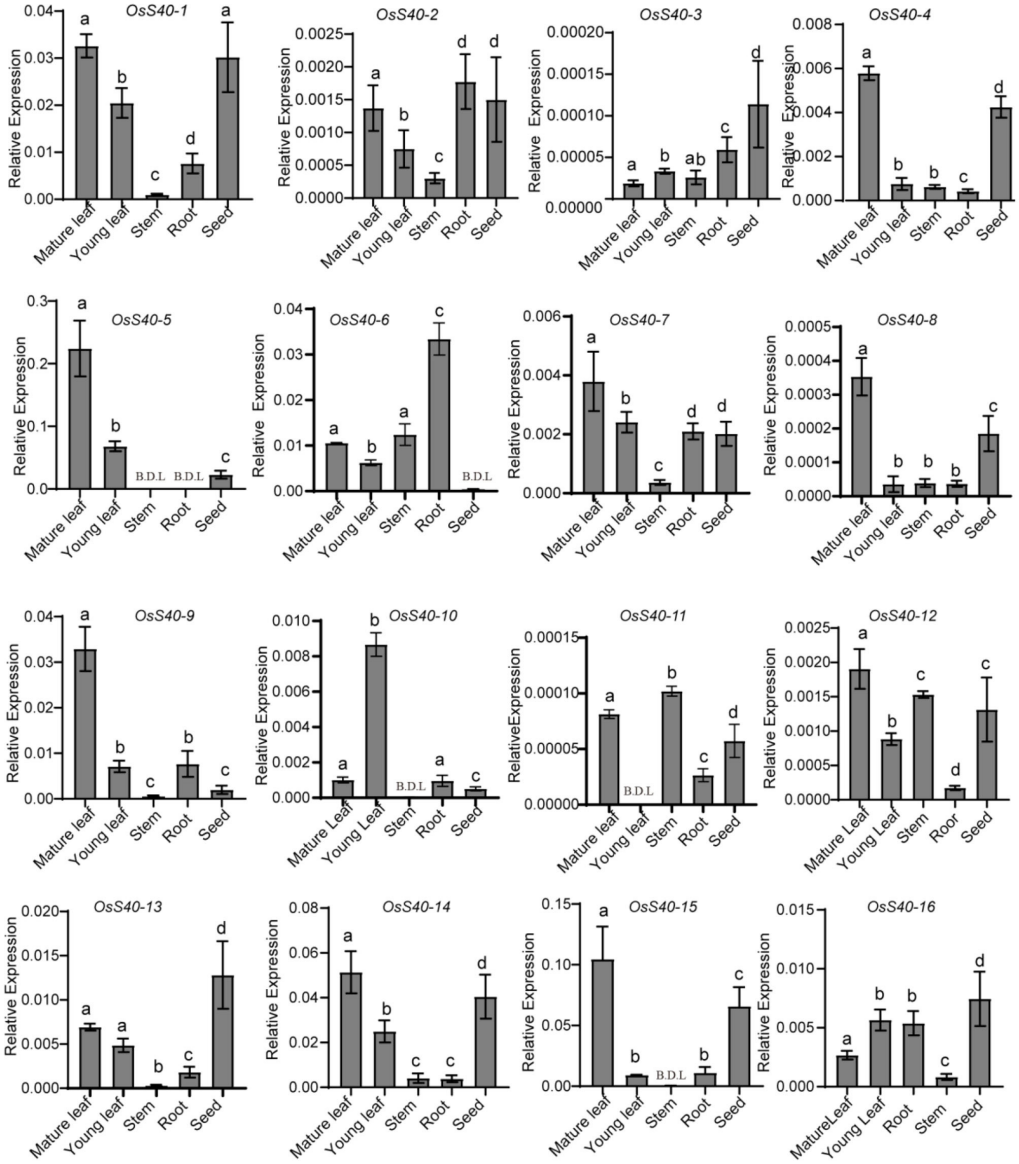
Tissue-specific expression of the *OsS40* gene family in rice. Transcript levels are expressed relative to rice *OsACTIN* in each sample, and values are mean ± SD. Mean and SD values were obtained from three biological replicates and three technical replicates. Statistically significant difference was determined using a one-way ANOVA test (*p* < 0.05). Groups carrying the same letter show no significant difference, while a significant difference is found between groups carrying the different letters of a, b, c, or d. B.D.L., below the detection limit.

Wild-type plants, Nipponbare (NIP) (*Oryza sativa* L. ssp. japonica), growing under optimum conditions, undergo natural senescence, which is controlled by various tissues in development. To gain insight into the potential functions of *OsS40* genes during natural rice growth, qRT-PCR detected the expression changes of all 16 *OsS40* members in rice young leaves, mature leaves, roots, stems, and seeds. Although the absolute mRNA levels of some *OsS40* genes, such as *OsS40-3* and *OsS40-11*, were extremely low, their transcript tendency in different tissues was still comparable. Among them, 12 members (*OsS40-1, OsS40-2, OsS40-4, OsS40-5, OsS40-6, OsS40-7, OsS40-8, OsS40-9, OsS40-11, OsS40-12, OsS40-14*, and *OsS40-15*) displayed higher transcript levels in mature leaves, while their transcript level was lower in young leaves (Figure 1), suggesting they may be involved in the leaf natural senescence. However, half of them, *OsS40-1, OsS40-2, OsS40-3, OsS40-4, OsS40-12, OsS40-13, OsS40-14, OsS40-15*, and *OsS40-16*, exhibited high expression levels in mature seeds with an efficiency comparable to their expressions in mature leaves (Figure 1), hinting at their possible functions during leaf aging and seed maturation.

**Figure 2:**
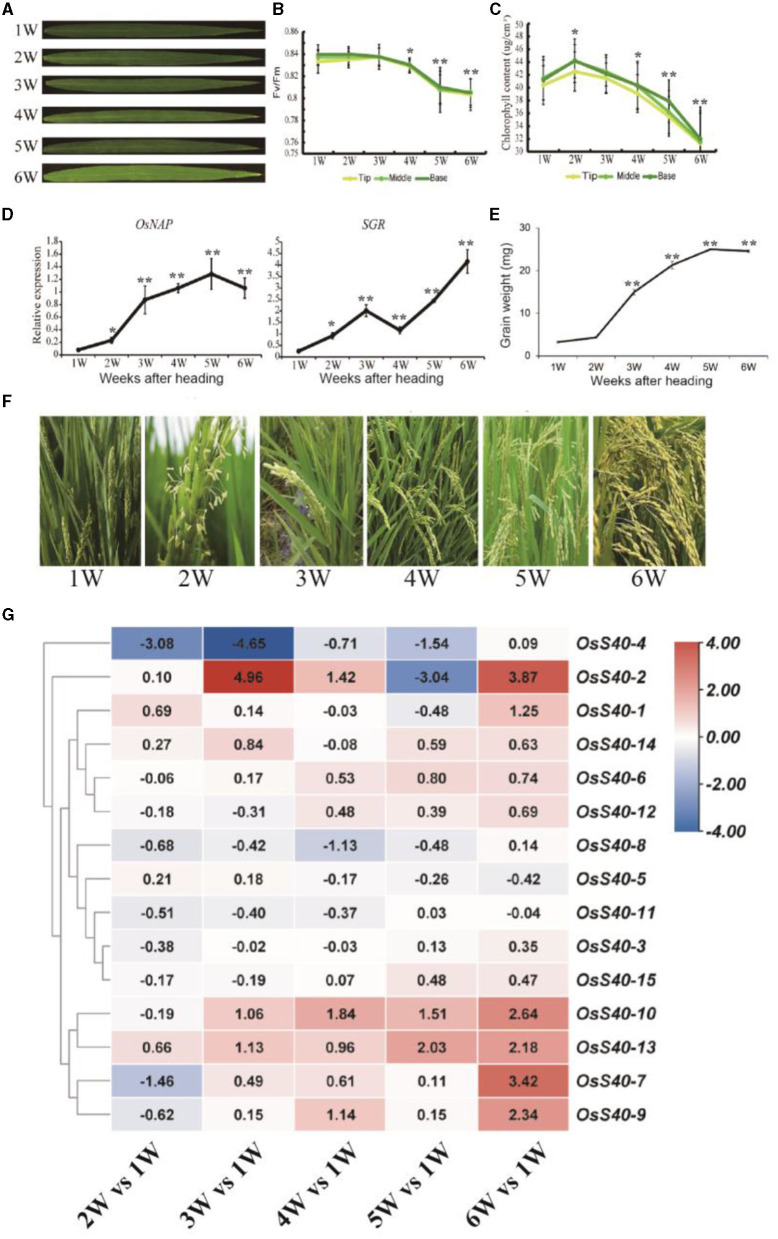
The expression profiles of the *OsS40* family from 1W (WAH) to 6W (WAH) during rice flag leaf aging **(A)**. The flag leaves during rice aging **(B,C)**. Photosystem II fluorescence efficiency (Fv/Fm) and chlorophyll contents of flag leaves during rice aging **(D)**. The senescence-related gene expression profiles of flag leaf during rice aging **(E)**. The grain weight during rice plant aging **(F)**. The whole plant of rice after heading during rice aging from 1W to 6W **(G)**. The expression profiles of the *OsS40* family during rice flag leaf aging; values were the RPKM ratio normalized to 1W; mean and SD values were obtained from three biological replicates. Asterisks indicate significant differences (^*^p < 0.05; ^**^p < 0.01) based on Student's *t*-test compared with 1W.

Rice flag leaves play a vital role in providing photosynthetic nutrients to young panicles during the grain-filling time frame and thus serve as an indicator of grain yield (Yoshida, 1972). To investigate the transcriptional changes during the aging of rice flag leaves and grain-filling, a temporal genome-wide transcriptome analysis was carried out in flag leaf tissue of the wild-type NIP through massive RNA sequencing as indicated in our previous research (Li et al., 2021). At the heading stage, the flag leaf was fully expanded at 1 week after heading (WAH), and its chlorophyll content, after reaching a peak at 2 WAH, gradually decreased from 2 WAH to 6 WAH, which was accompanied by a smooth decline in photochemical efficiency (Figures 2A–C). Flag leaf samples were collected six times with a 1 week interval: 1 WAH, 2 WAH, 3 WAH, 4 WAH, 5 WAH, and 6 WAH. The onset of leaf senescence coincides with the start of chlorophyll (Chl) degradation, while the initiation of leaf senescence is before Chl degradation. Therefore, the senescence initiation of flag leaves started at the time period between 1 WAH and 3 WAH (Li et al., 2021). Two senescence-associated marker genes, *OsNAP* and *SGR*, also showed an increased expression level at the early aging stage of flag leaves (Figure 2D). At the same stage, grain weight was measured and found to be significantly increased from 2 WAH up to the peak at 5 WAH, and then kept stable at 6 WAH, indicating grain-filling initiation at 2-3 WAH (Figures 2E,F).

The transcriptome data from different flag leaf senescence stages revealed that 6 of the 16 members, *OsS40-1, OsS40-2, OsS40-7, OsS40-9, OsS40-10*, and *OsS40-13*, accumulated 2- to 4-fold higher transcript levels at the late stage of flag leaf senescence (Figure 2G; Supplementary Dataset 1). These results demonstrated that the *OsS40* gene expressions are closely related to leaf natural senescence and seed development.

### Identification of *Oss40* Mutants Involved in Flag Leaf Senescence and Grain Weight

**Figure 3:**
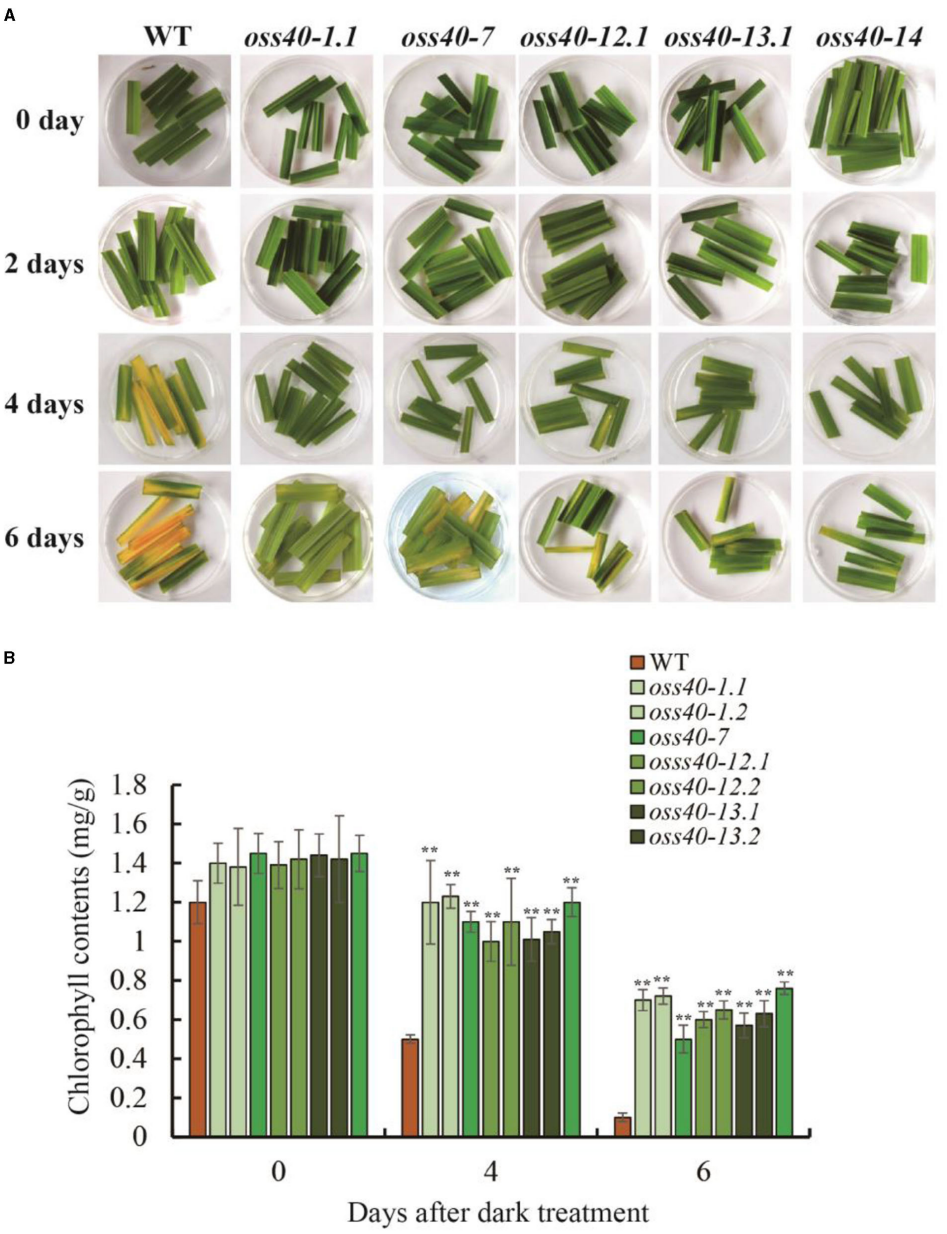
The stay-green phenotype of the detached leaves of different *oss40* mutant lines after darkness treatment. **(A)** The detached leaves from three plants represent the stay-green phenotype of *oss40* (−1, −7, −12, −13, and −14) mutants relative to WT, respectively, at 0, 2, 4, 6 days after darkness treatment. **(B)** The total chlorophyll levels of WT and *oss40* mutants at 0, 4, 6 days after darkness treatment, respectively. Ten pieces of detached leaves were used for chlorophyll extraction and content measurement. Mean and SD values were obtained from three biological replicates. Significant differences of the chlorophyll levels normalized to WT were evaluated using Student's *t*-test (^**^p < 0.01).

To genetically investigate the biological roles of *OsS40* genes in plant growth and leaf senescence, CRISPR/Cas9 gene-edited technology was employed to generate knockout mutant lines of the *OsS40* gene family in rice. CRISPR/Cas9-edited lines of 13 *OsS40* members were obtained, and the mutations in the targeted exon regions were identified by genomic DNA sequencing. They are mostly characterized by short nucleotide insertions or deletions near their respective sgRNA sequences, leading to ORF shifts in the *OsS40* genes (Table 1). Based on *OsS40* gene expression profiles in rice flag leaves mentioned above, we expected the knockout of *OsS40* members would affect leaf senescence. We did not observe visible phenotypic differences in primary leaf or seedling development between these *oss40* mutants and the wild type (NIP) at 4-week-old seedling under greenhouse conditions. However, when the detached primary leaves were incubated for 4–6 days under darkness treatment, the delayed leaf senescence occurred in the five *OsS40* member knockout lines (*oss40-1, oss40-7, oss40-12, oss40-13*, and *oss40-14*) relative to wild-type (Figure 3A); consistently, the chlorophyll contents were maintained at 70–80% at 4 days after dark treatment and maintained at 50–60% at 6 days after dark treatment in the *oss40-1, oss40-7, oss40-12, oss40-13*, and *oss40-14* mutants relative to wildtype (Figure 3B), suggesting that *OsS40*-1, *OsS40*-7, *OsS40*-12, *OsS40*-13, and *OsS40*-14 are involved in the dark-induced senescence pathway.

**Figure 4:**
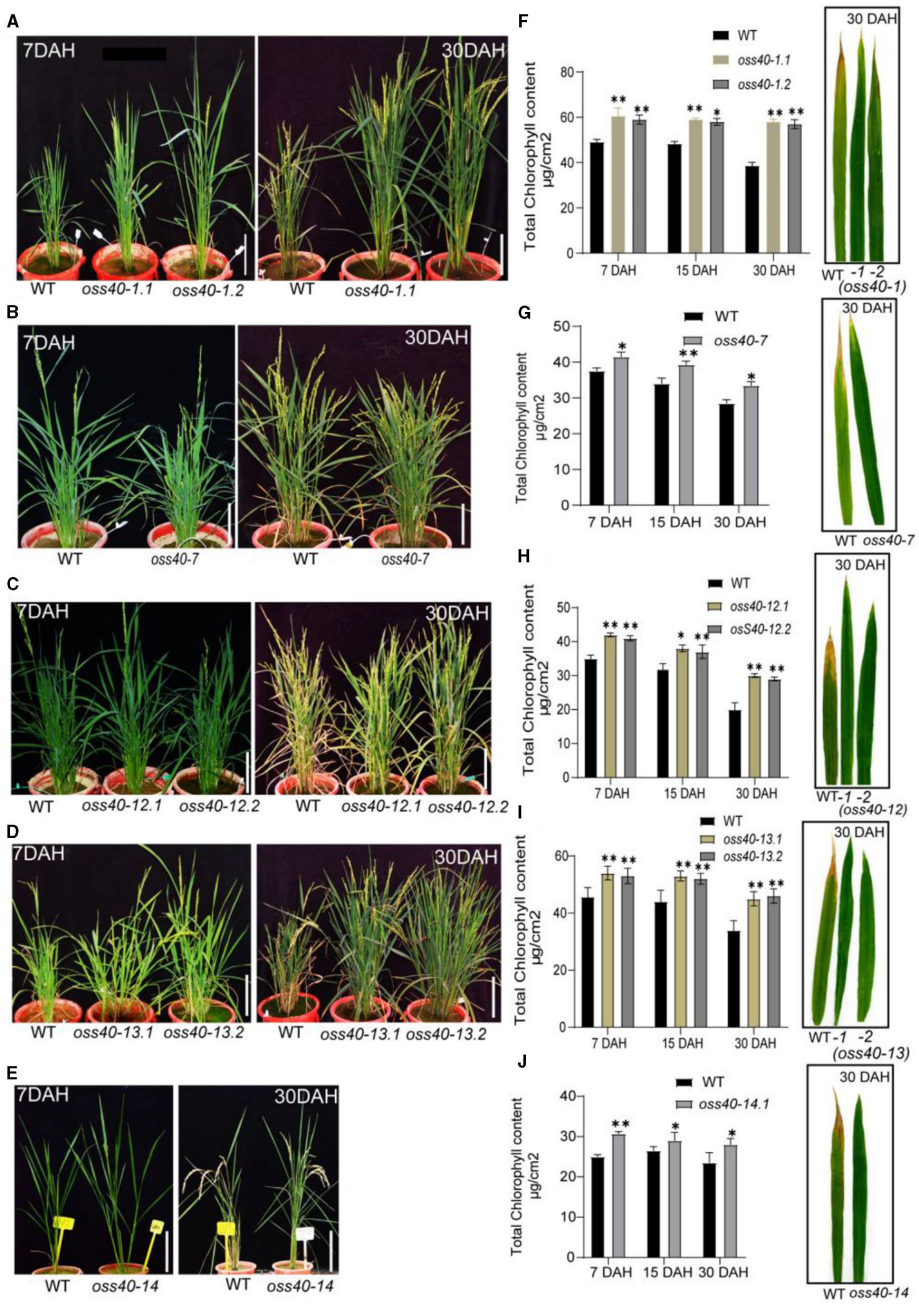
The stay-green phenotype of different *oss40* mutant lines during natural senescence. **(A,C,E,G,I)** represents the phenotype of WT and *oss40* (−1, −7, −12, −13, and −14) mutants, respectively, at 7 DAH and 30 DAH (days after heading). **(B,D,F,H,J)** show total chlorophyll levels of WT and *oss40* mutants at 7DAH, 15DAH, and 30DAH, respectively. Five flag leaves were used for chlorophyll content measurement. Mean and SD values were obtained from three biological replicates. Significant differences of the chlorophyll levels normalized to WT were evaluated using a paired Student's *t*-test (^*^p < 0.05; ^**^p < 0.01).

Rice flag leaf, which is the last leaf appearing before the inflorescence, is commonly considered as the main source of photosynthetic products for the panicle and is correlated with panicle weight, grain filling, and other yield-related features (Cui et al., 2003; Mei et al., 2003; Zahid et al., 2006; Tang et al., 2011). To further evaluate the impact of *OsS40* genes on the natural senescence and plant senescence of flag leaves, we scrutinized age-related flag leaf yellowing in the *oss40* mutants and NIP growing under the same growth conditions. We screened and observed the plant phenotypes of all *oss40* mutants compared with the NIP plant. Interestingly, a stay-green phenotype was observed in five *OsS40* member knockout lines (*oss40-1, oss40-7, oss40-12, oss40-13*, and *oss40-14*), at 30 days after heading (DAH). In comparison to NIP, greater plant height occurred in the *oss40-1, oss40-13*, and *oss40-14* mutants, and more tillers appeared in the *oss40-13* mutants at 7 DAH (Figures 4A–E). The chlorophyll contents of flag leaves from 10 plants per line at 7, 15, 30 DAH were measured and found to be significantly increased in the *oss40-1* (0.1, 0.2), *oss40-7.1, oss40-12* (0.1, 0.2), *oss40-13* (0.1, 0.2), and *oss40-14.1* mutants at 30 DAH compared with NIP (Figure 4G), which was consistent with the delayed flag leaf yellowing phenotype of one representative flag leaf of 10 plants (Figure 4H). According to chlorophyll content quantification, the *oss40-1, oss40-7, oss40-12, oss40-13*, and *oss40-14* mutants appeared to have a stay-green phenotype compared with NIP (Figure 4). These findings showed that *OsS40*-1, *OsS40*-7, *OsS40*-12, *OsS40*-13, and *OsS40*-14 might positively impact leaf senescence in rice.

**Figure 5:**
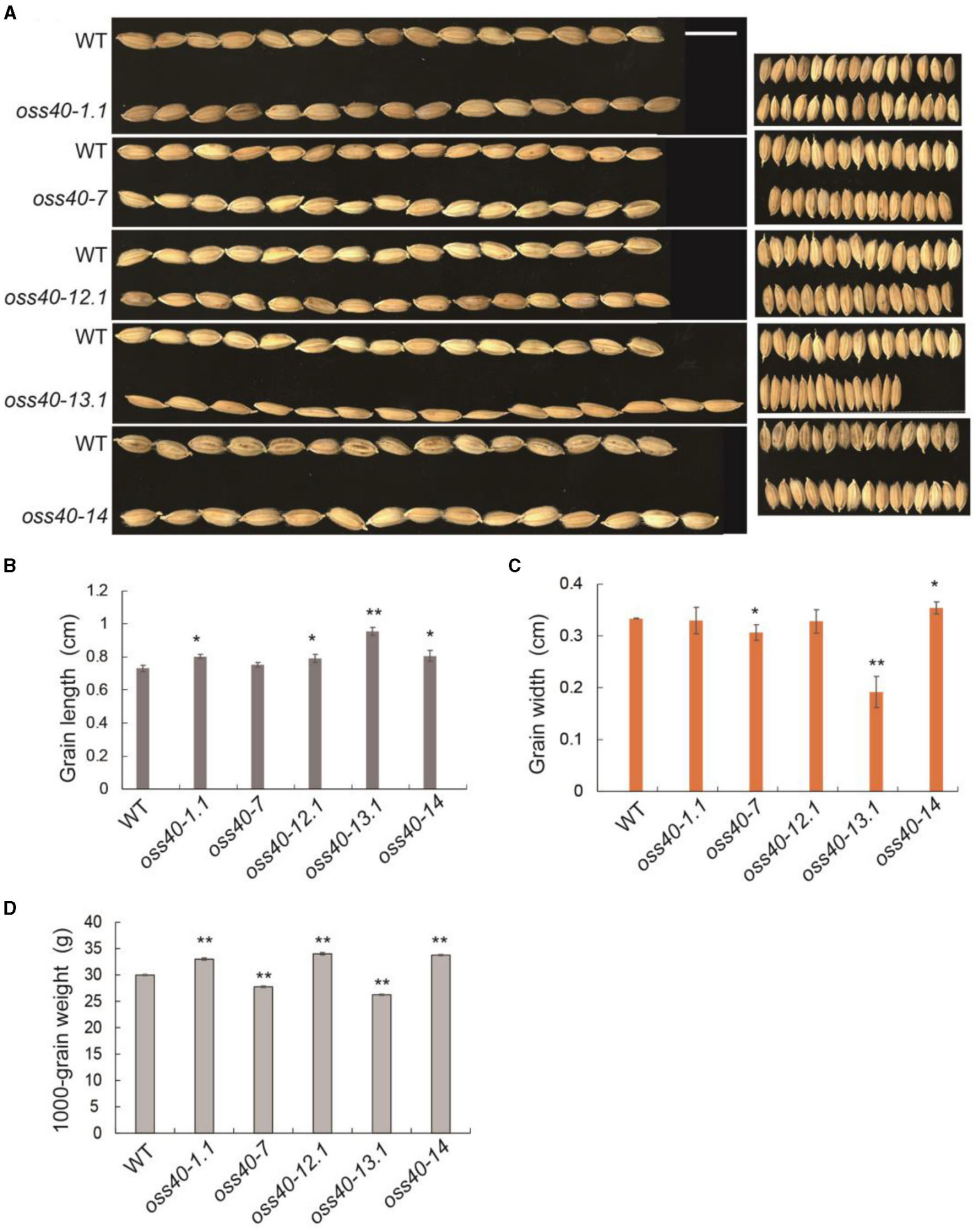
Grain size and grain weight phenotypes of different *oss40* mutant lines compared with WT (NIP) **(A)**. A representative grain phenotype of various *oss40* mutants relative to WT. Scale bar = 10 mm **(B,C)**. The grain length **(B)** and width **(C)** of 20 grains were randomly taken from various *oss40* mutants relative to WT **(D)**. About 1,000-grain weight of various *oss40* mutants relative to WT. The values are mean ± SD. Mean and SD values were obtained from three biological replicates and three technical replicates. Significant differences of the values normalized to WT were evaluated using Student's *t*-test (^*^p < 0.05; ^**^p < 0.01).

Besides considering the potential contribution of flag leaf senescence on yield-related features, grain size and grain weight were investigated among the stay-green *oss40* mutants relative to NIP. The grain width of the *oss40-7* and *-13* mutant was narrower than that of NIP, leading to a decreased grain weight, while grain length of the *oss40-1* and -*14* mutants was much longer relative to NIP, resulting in an increased grain weight (Figures 5A–D).

### Altered Expressions of SAGs and CCGs in *OsS40* Family Mutants

**Figure 6:**
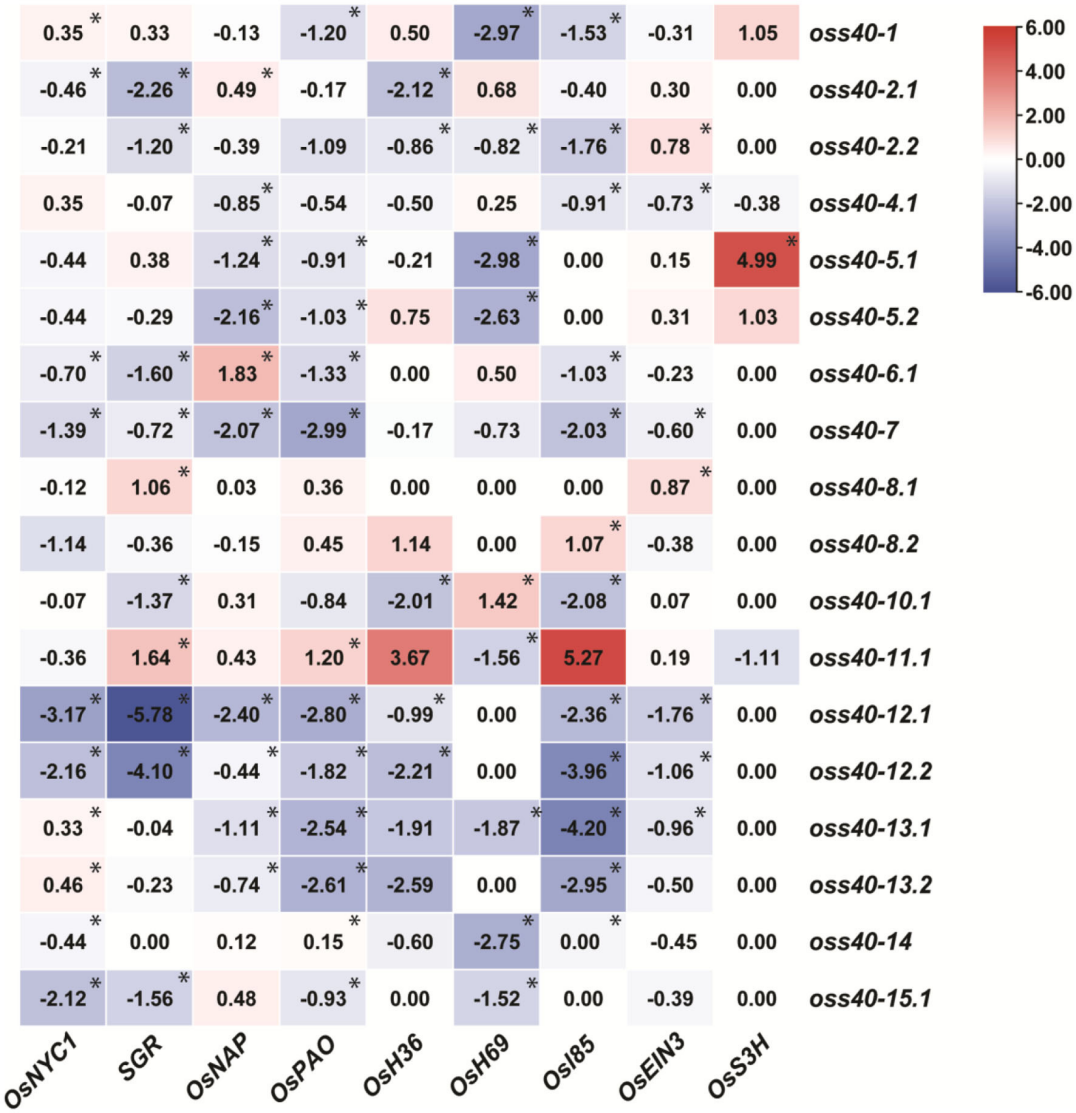
Altered expression of CDGs and SAGs in different *oss40* mutant lines. Heatmap representation shows the CDGs (*OsNYC1, SGR*, and *OsPAO*) and SAGs (*OsH36, OsH69, Os185, OsNAP, OsEIN3*, and *OsS3H*) expression level in different mutant lines. The expression levels of genes are presented using fold-change values log2 format. The data indicate the relative expression levels normalized to that of the internal control *OsACTIN*. Red and blue colors correspond to up- and down-regulation of the gene expressions, respectively. Mean and SD values were obtained from three biological replicates. Significant differences of the expression levels normalized to WT were evaluated using Student's *t*-test (^*^p < 0.05).

We further detected gene expression changes of different kinds of senescence-related genes. To this end, first, we selected several kinds of senescence marker genes. For example, chlorophyll degradation genes (CDGs), such as Os*NYC1, OsSGR*, and *OsPAO*, and senescence-associated genes (SAGs), such as glyoxylate aminotransferase (*OsH36*), seed imbibition protein (*OsH69*), and isocitrate lyase (*Osl85*). Various hormonal-signaling pathway-related genes such as ABA signaling-related *OsNAP*, ethylene signaling-related ETHYLENE INSENSITIVE 3 (*OsEIN3*), and SA signaling-related SALICYLIC ACID 3 HYDROXYLASE (*OsS3H*). The transcript levels of several SAGs and CDGs in senescing mature leaves (the first completely developed leaf from the bottom at the four-leaf stage) of *oss40s* knockout mutants and NIP were examined by qRT-PCR. The data indication by heatmap analysis revealed that most of the tested senescence-related marker genes were significantly downregulated in *oss40-7, oss40-12*, and *oss40-13* mutants compared with NIP, while a few of them showed higher expression in *oss40-8* and *oss40-11* mutants. The transcript levels of these SAGs and CDGs showed a different but declining tendency in the remaining *oss40* mutants, except for the transcript level of *OsS3H*, which exhibited clear upregulation in the *oss40-5* mutants (Figure 6; Supplementary Figure 1), suggesting that these *OsS40* members may play unique roles in various senescence-associated pathways.

### Altered Expressions of *OsWRKY* and *OsSWEET* Genes in the Stay-Green *oss40s* Mutants

**Figure 7:**
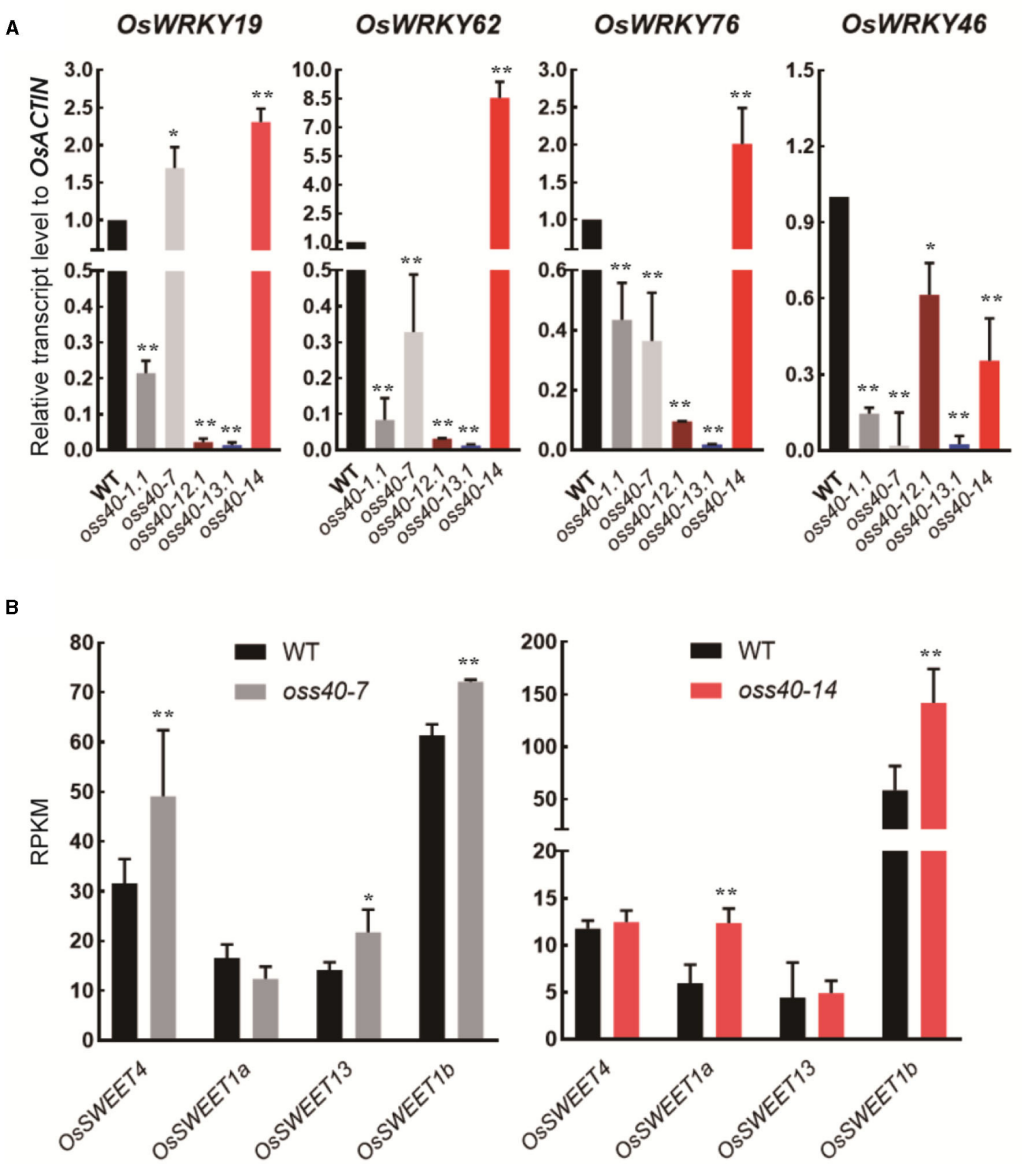
Expression profiles of selected WRKY members and SWEET members in the stay-green *oss40* mutants relative to WT **(A)**. Expression profiles of selected WRKY members (*OsWRKY19, OsWRKY62, OsWRKY76*, and *OsWRKY46*) in five stay-green *oss40* mutants relative to WT **(B)**. Expression profiles (RPKM) of selected SWEET members (*OsSWEET1a, OsSWEET1b, OsSWEET4*, and *OsSWEET13*) in the *oss40-7* and *oss40-14* mutants relative to WT based on the RNA-seq dataset of mutant and WT leaves at 77 days after sowing. The values are mean ± SD. Mean and SD values were obtained from three biological replicates. Significant differences of the expression levels normalized to WT were evaluated using Student's *t*-test (^*^p < 0.05; ^**^p < 0.01).

To detect whether WRKY members are the downstream genes of *OsS40*s, transcript levels of these senescence-related *WRKY* members, selected from the DEGs of *oss40-14* /WT RNA-seq dataset (Supplementary Dataset 2), were monitored in fully developed leaves of *oss40-1, oss40-7, oss40-12, oss40-13*, and *oss40-14* relative to WT by qRT-PCR. All four of the tested *WRKY* genes (*WRKY19, WRKY62, WRKY76*, and *WRKY46)* were downregulated in the *oss40-1, oss40-12*, and *oss40-13* mutants. With the exception of *WRKY19*, the other *WRKY* genes showed decreased expression levels in the *oss40-7* mutant. However, only *WRKY46* displayed reduced transcript levels in the *oss40-14* mutant (Figure 7A). This result suggested that WRKY members related to rice leaf senescence might be positively regulated by the five stay-green associated *OsS40* genes.

Based on the grain size and grain weight phenotype in the stay-green *oss40-7* and *oss40-14* mutants, we supposed *OsS40*-7 and *OsS40*-14 may affect source and sink carbon distribution during leaf aging. To test this possibility, we detected expression changes of the sugar transporters *OsSWEETs* gene family members, which were selected from the DEGs of *oss40-7/*WT and *oss40-14*/WT RNA-seq dataset (unpublished dataset). The expression levels of *OsSWEET4, OsSWEET1a, OsSWEET13*, and *OsSWEET1b* were altered in the 1-month-old fully expanded primary leaves of both *oss40-7* and *oss40-14* mutants compared with WT. *OsSWEET4, OsSWEET13*, and *OsSWEET1b* were upregulated in the *oss40-7* mutant, while *OsSWEET4, OsSWEET1a*, and *OsSWEET1b* were upregulated in the *oss40-14* mutant (Figure 7B). It seems that stay-green flag leaves and the high-grain weight phenotype in the *oss40-14* are consistent with a stay-green phenotype in the *ossweet4* mutant, while the low-grain weight phenotype in the *oss40-7* is consistent with a dry grain phenotype in the *ossweet13* mutant (Sosso et al., 2015; Bezrutczyk et al., 2018; Yang et al., 2018). Therefore, *OsS40*-7 and *OsS40*-14 might be negative regulators of different OsSWEET members, respectively, involving in the regulation of sucrose transportation and grain filling.

### Subcellular Localization and Transcriptional Regulation Activity of the Five “Stay-Green” *OsS40* Members

**Figure 8:**
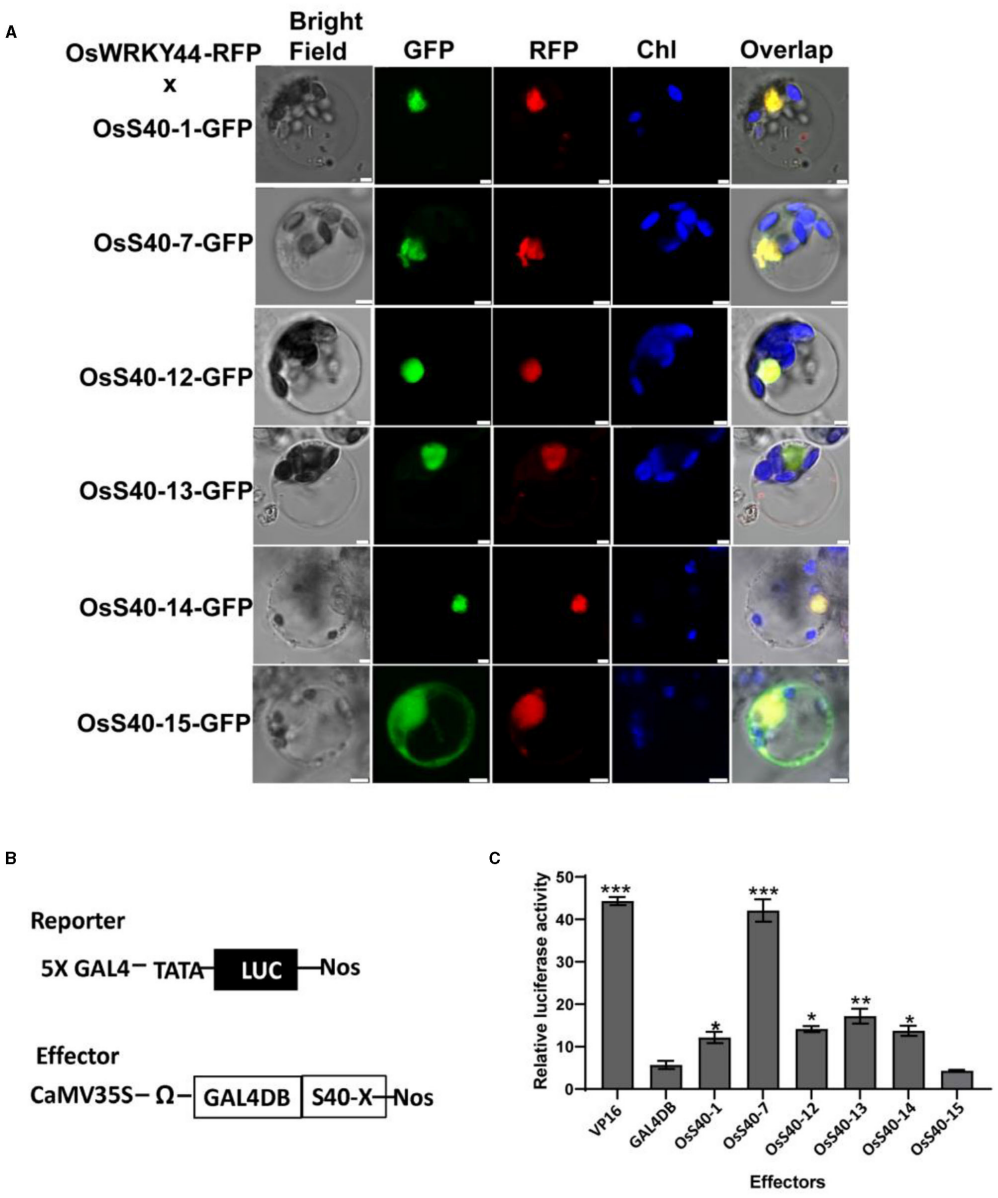
Subcellular localization and transcriptional regulation activity of *OsS40*. **(A)** Subcellular co-localization of *OsS40*s-GFP and OsWRKY44-RFP proteins in rice protoplasts as revealed by green fluorescence and red fluorescence, respectively. Scale bar = 5 μm **(B)** A scheme of reporter and effector constructs used in the transcriptional regulation assay. The GAL4-responsive reporter constructs, GAL4-LUC, containing five copies of the GAL4-binding site in tandem and a minimal TATA region (starting at position 46) of the CaMV 35S promoter, the firefly gene for luciferase (LUC), and a nopaline synthase (nos) terminator. Each effector construct contained a GAL4 DNA-binding domain (GAL4DB) and a coding region of *OsS40* driven by the CaMV 35S promoter. **(C)** Effects of the *OsS40* on reporter gene expression as revealed by relative LUC activity. The GAL4 DNA-binding domain (DB) and VP16 were used as negative and positive controls, respectively. Mean and SD values were obtained from three biological replicates. Significant differences of the LUC levels normalized to GAL4 DB were evaluated using Student's *t*-test (^*^p < 0.05; ^**^p < 0.01, ^***^p < 0.001).

It has been reported that *OsS40* members are distributed in different subcellular compartments, including the nucleus, cytoplasm, or some unknown speckles in the cytoplasm (Zheng et al., 2019). To further clarify the subcellular localization of five stay-green *OsS40* members, C-terminal GFP-tagged *OsS40*-1, *OsS40*-7, *OsS40*-12, *OsS40*-13, and *OsS40*-14, were co-expressed with a nuclear protein, OsWRKY44-RFP, in rice protoplasts, since the nucleus-targeting property of OsWRKY44-GFP has been confirmed by DAPI staining (Supplementary Figure 2). Laser scanning confocal microscopy imaging showed that all five *OsS40* proteins were predominantly accumulated in the nucleus, as illustrated by the overlap of the GFP and RFP fluorescence signals (Figure 8A). At the same time, the *OsS40*-15 was visualized in both the nucleus and the cytoplasm.

The putative DNA-binding property of *OsS40* members was highlighted with web-based applications SVMProt-like nuclear-localized HvS40 and AtS40–3 (Cai et al., 2003; Li et al., 2016). Thus, we deduced these nuclear-localized *OsS40* proteins could serve as transcription factors or regulators. We examined the transcriptional activation activity of these *OsS40* members using dual-luciferase (LUC) activity assays in the rice protoplast system (Figure 8B). As shown in Figure 8C, GAL4DB-fused *OsS40*-1, *OsS40*-7, *OsS40*-12, *OsS40*-13, and *OsS40*-14 were able to significantly induce the reporter LUC activity, in contrast to the negative BD control. The *OsS40*-15 mirrored the result obtained in the negative BD control and thus appeared not to have such activation activity. In particular, *OsS40*-7 dramatically promoted reporter gene activity with an efficiency comparable to the positive VP16 control. These results support that the five stay-green *OsS40* members possess transcriptional regulation activity.

### Transcript Profiles of **OsS40** Family Genes in the *Oss40s* Mutants

Since most *OsS40* family genes were induced differentially during leaf senescence (Figure 2; Zheng et al., 2019), and five stay-green *oss40* mutants have a similar phenotype and all appear to have transcriptional regulation activity, we assumed that *OsS40* genes could be associated in a family network to regulate themselves and downstream SAGs expression in leaf senescence network. To achieve some molecular clues about the relationships of *OsS40* genes in the *OsS40* family-dependent regulatory network, expression patterns of the 16 *OsS40* genes were examined with senescing mature leaves of 30 *oss40s* mutants and NIP by qRT-PCR.

Heatmap analysis revealed that seven and five *OsS40* genes were significantly downregulated in the *oss40-12* and *oss40-7* mutants, respectively, whereas six and eight *OsS40* genes were upregulated in the *oss40-13* and *oss40-14* mutants, respectively (Supplementary Figure 3). These results indicated complex relationships among these *OsS40* members. However, except for *OsS40-1, OsS40-7, OsS40-12, OsS40-13*, and *OsS40-14*, a branch of *OsS40* genes, including *OsS40-2, OsS40-8, OsS40-9, OsS40-15*, and *OsS40-16*, displayed statistically significant alteration of expression in at least three stay-green *oss40s* mutants, suggesting these 10 members might be functionally related and involved in natural senescence of the rice leaf.

**Figure 9:**
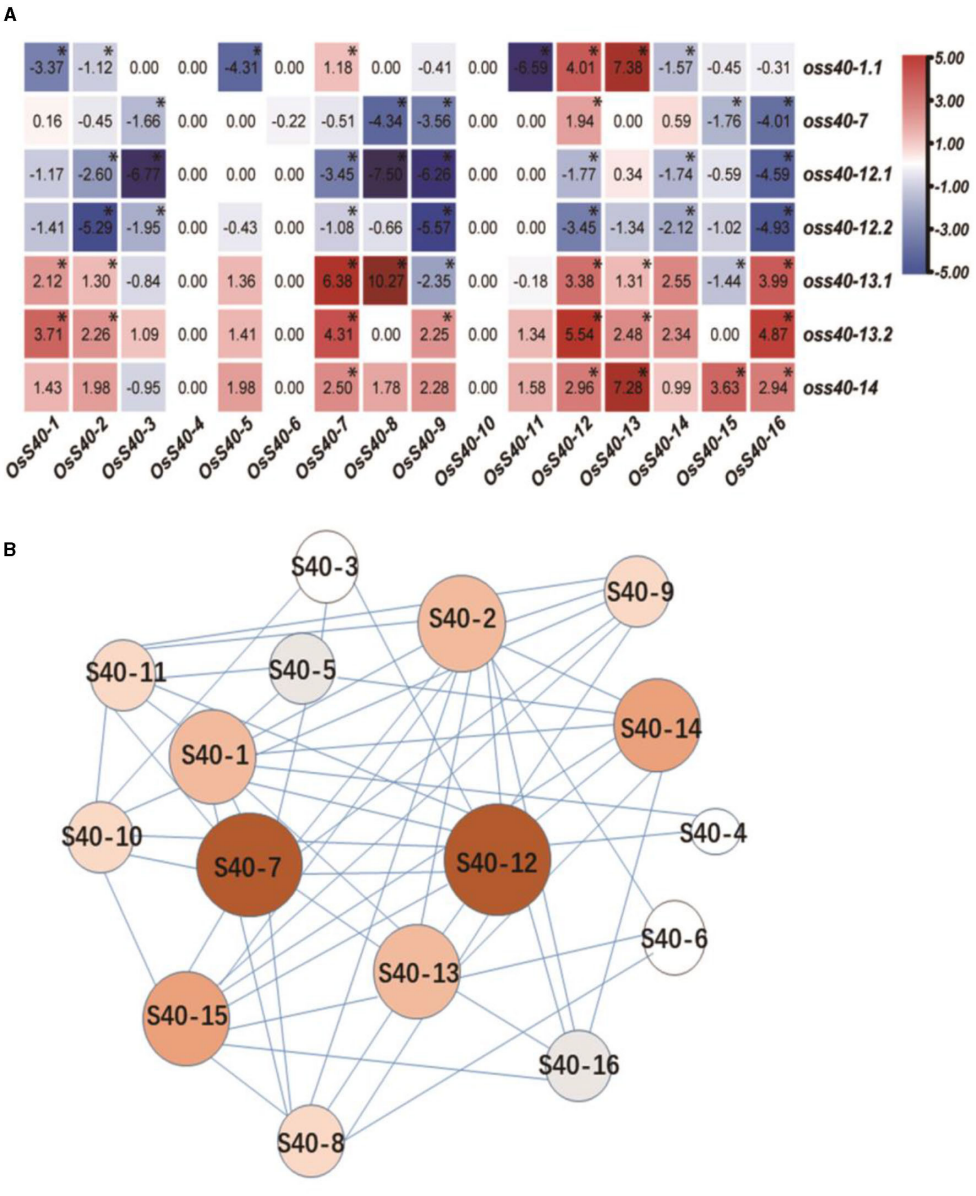
The co-expression and regulatory network inside the S40 family. **(A)** Heatmap representation shows *OsS40* expression levels in *oss40* (−1, −7, −12, −13, and −14) mutant lines. The expression levels of genes are presented using fold-change values log2 format. The data indicate the relative expression levels normalized to that of the internal control *OsACTIN*. Red and blue colors correspond to up- and down-regulations of the gene expressions, respectively. Mean and SD values were obtained from three biological replicates. Significant differences of the expression levels normalized to WT were evaluated using Student's *t*-test (^*^p < 0.05). **(B)** Structure of the co-expression network in the S40 family. In this network, the red nodes represent more associations with other members; the white nodes represent fewer associations with other members, indicating the alteration of other member gene expression in one mutant.

To visualize the connections among these *OsS40* genes based on their differential expressions in the *oss40* mutants, we ran a regulation network program, which was modified from TimeXNet Web (http://txnet.hgc.jp/). With a threshold of *p* < 0.05, the generated hub network revealed that *OsS40*-7 and *OsS40*-12 presented most associations with other *OsS40* members, which was reflected by the biggest size and darkest red in their nodes. It implied that *OsS40-7* and *OsS40-12* may act as the master members controlling the expression of other natural senescence-related *OsS40* genes (Figure 9).

## Discussion

The DUF584 domain is one of over 3,000 domains of unknown function (DUF) families in the Pfam database. Currently, there are 201 DUF584 domain-containing proteins listed (Bateman et al., 2000). Although very little research has been conducted on DUF584 proteins to date, work on HvS40, a DUF584-harboring protein in barley, suggests a key role in barley senescence. A recent finding has shown that AtS40-4 with the DUF584 motif functioned as a negative regulator in ABA signaling and affected seed germination and seedling growth of *Arabidopsis* under ABA treatment (Shi et al., 2021). Based on the HvS40 protein sequence, 16 *OsS40* members were identified from the rice genome database and characterized by differential upregulation during natural or stress-induced leaf senescence (Zheng et al., 2019). In this study, we generated and characterized five *oss40s* (*oss40-1, oss40-7, oss40-12, oss40-13*, and *oss40-14*) mutants, showing a stay-green phenotype compared with wild type; a similar phenotype was noted in the *A. thaliana* T-DNA insertion mutant line of *ats40-3* (Fischer-Kilbienski et al., 2010). These five senescence-related members of the *OsS40* gene family all have transcriptional activation activity and subcellular localization in the nucleus. Meanwhile, epitopic genetic analysis revealed complex relationships among *OsS40* members. These findings suggest that these five *OsS40* members might play a critical role during natural leaf senescence conditions.

Several studies have revealed how grain filling and grain weight of cereal crops are strongly affected by premature leaf senescence. Over 50% of the carbon content in grains is contributed by remobilized storage carbohydrates in flag leaves (Gladun and Karpov, 1993; Yoo et al., 2007; Gregersen et al., 2013). Thus, premature leaf senescence may limit photosynthate assimilation and grain filling. On the contrary, rice production may be improved by delaying leaf senescence appropriately (Liang et al., 2014; Mao et al., 2017). The higher expression of *OsS40* genes in the later stage of flag leaf senescence and comparatively higher expression in mature leaves (Figures 1, 2) may correlate the *oss40* mutants with flag leaf senescence and rice grain productivity. This may explain why the loss-of-function mutants of these five *OsS40s* did not show phenotypic differences compared with wild type in the primary leaf and seedling stages but showed stay-green phenotype and high/low biomass compared with wildtype, which is consistent with rice *ossweet4, ossweet13*, and *ossweet11/15* mutants (Sosso et al., 2015; Yang et al., 2018). It is well-known that sugar transporters of the SWEET family play vital roles in the sink-source relationship and plant reproductive development. Actually, in this study, the expression levels of *OsSWEET4, OsSWEET1a, OsSWEET13*, and *OsSWEET1b* were altered in both *oss40-7* and *oss40-14* mutants (Figure 7B). *OsSWEET4* had been shown to encode a hexose transporter, and its null mutants appeared a strong empty pericarp (emp) phenotype in developing rice grains and stay-green spikes (Sosso et al., 2015). While ZmSWEET13a, b, c, the orthologs of OsSWEET13 in maize, can mediate sucrose transport and are essential for efficient phloem loading, accumulation of starch and soluble sugar, as well as accelerated senescence, was observed in leaves of the triple *zmsweet13* knockout mutants (Bezrutczyk et al., 2018). It has been proposed that AtSWEET13 functions as a “revolving door” mechanism accelerate transport efficiency (Feng and Frommer, 2015; Han et al., 2017; Latorraca et al., 2018). OsSWEET1a/1b is currently unknown; AtSWEET1 in *Arabidopsis* is a plasma membrane hexose transporter (Chen et al., 2010; Tao et al., 2015). *OsS40*-7 and *OsS40*-14 might affect sucrose transportation and grain filling as upstream regulators of OsSWEETs.

As known, in senescing leaves, chlorophylls are sequentially degraded by chlorophyll catabolic enzymes (CCGs) through upregulation of CDGs, such as *OsNYC1* (Kusaba et al., 2007), *OsSGR* (Park et al., 2007), and *OsPAO* (Tang et al., 2011). Many other SAGs are upregulated during natural and dark-induced senescence in rice, with products identified as glyoxylate aminotransferase (*OsH36*), seed imbibition protein (*OsH69*), and isocitrate lyase (*Osl85*) (Lee et al., 2001). Various hormonal signaling pathway-related genes, such as ABA signaling-related *OsNAP* (Liang et al., 2014), ethylene signaling-related ETHYLENE INSENSITIVE 3 [*OsEIN3* (Mao et al., 2006)], and SA signaling-related SALICYLIC ACID 3 HYDROXYLASE [*OsS3H* (Zhang et al., 2013)] are involved in the regulatory pathway of leaf senescence (Luoni et al., 2019). Therefore, in this study, although downregulation of some CDGs or SAGs also occurred in the other *oss40s* (*oss40-2, oss40-4, oss40-5, oss40-6, oss40-8, oss40-10, oss40-11*, and *oss40-15*) mutant lines, the stay-green phenotype did not appear in their senescencing flag leaves. However, after dark treatment, the detached leaves appear to delay senescence in the five mutants, which possibly hinder progression of natural senescence and improve vegetative growth, leading to delayed flag leaf yellowing or higher plant height or more tillers. For example, rice *osein2/mhz7* mutant lines showed delayed dark-induced leaf senescence and higher plant height under field-grown conditions (Ma et al., 2013). This is, perhaps, not surprising since these mutants may not only be involved in age-specific developmental senescence but also in response to environmental stress-mediated senescence due to the differentially induced transcript levels of these *OsS40* genes under various conditions of senescence (Zheng et al., 2019). It has been demonstrated that mutation of *OsSRLK*, encoding a senescence-induced receptor-like kinase, or mutation of *OsRL3*, encoding an MYB-related TF, results in a stay-green phenotype in detached leaves during dark-induced senescence, but no phenotypic differences were observed between wild type and the two mutants during natural senescence under field condition (Park et al., 2018; Shin et al., 2020). Using the identified *oss40s* mutants in this study, the potential roles of *OsS40* members during abiotic or biotic stress-dependent senescence would be expected.

Transcription factors often function as connectors in gene regulatory networks, and, in some cases, a single transcription factor (TF) can even control an entire cellular process (Guo and Gan, 2014). Therefore, TF related to SAGs frequently plays a vital role in controlling leaf senescence, such as AtWRKY53-controlling leaf senescence *via* a very complex regulatory network (Robatzek and Somssich, 2002; Miao et al., 2004; Liu et al., 2016). It is documented that AtS40-3 acts as an upstream regulator of *AtWRKY53* (Miao et al., 2004) and the senescence-related genes *SAG12* and *SEN1* (Fischer-Kilbienski et al., 2010). In this study, the five crucial stay-green *OsS40* proteins were shown to have transcriptional activation activity (Figure 8), implying that these *OsS40* proteins might function as TFs, modulating a branch of senescence-related genes. *OsS40*-5 can target the promoter region of wheat *TaWRKY53* in a yeast one-hybrid screen (Van Eck et al., 2014). Our results also showed several senescence-associated rice WRKY members were differentially downregulated in the five stay-green *oss40* mutants (Figure 7A). It has been reported that the rice WRKY19, WRKY62, WRKY76, and WRKY46 were highly expressed in the senescent flag leaves (Li et al., 2021). OsWRKY62 and OsWRKY76 can form heterocomplexes and negatively regulate plant defense response (Liu et al., 2016). The expression of OsWRKY46 also dramatically induced iron stress or nitrogen starvation (Yang et al., 2016; Viana et al., 2017). Rice WRKY19 was involved in rice immunity mediated by the OsMKK4-OsMPK3/6 cascade (Kim et al., 2012). It suggests that *OsS40*s are involved in WRKY family regulation, which further supports our hypothesis that *OsS40* members act as a novel TF family in the senescence regulatory network. Furthermore, identifying downstream targets of *OsS40*s would be an important objective.

To uncover the possible relationships among these *OsS40* genes during developmental senescence, we observed the expression of 16 *OsS40s* members in the five stay-green *oss40* mutant lines (Figure 8). *OsS40-7* and *OsS40-12* act upstream of the other *OsS40* genes, since nearly half of the tested *OsS40* genes showed decreased transcript levels in the *oss40-7* and *oss40-12* mutants. However, quite a few *OsS40* genes, including *OsS40-7* and *OsS40-12*, were strongly upregulated in the *oss40-13 and oss40-14* mutants. The possibility is that *OsS40*-7 and *OsS40*-12 could act functionally as redundancy of *OsS40*-13 or *OsS40*-14. With respect to transcript profiles of SAGs and *OsS40* family genes in the generated stay-green *oss40* mutants, *OsS40*-1, *OsS40*-7, *OsS40*-12, *OsS40*-13, and *OsS40*-14 are anticipated to be functionally correlated with rice developmental senescence by regulating other *OsS40* members and senescence-associated genes expression. Considering that many rice WRKY TFs are integrated into complicated signaling networks by forming homodimers or heterodimers (Viana et al., 2018), it would be interesting to perform interaction analysis of the *OsS40* gene family to elucidate their associations in more detail.

## Data Availability Statement

The datasets presented in this study can be found in online repositories. The names of the repository/repositories and accession number(s) can be found in the article/Supplementary Material.

## Author Contributions

XZ and YM conceived and designed the research. H, JX, AG, YL, CF, JU, and CH collected the data. H, JX, YZ, and XZ analyzed the data. H, XZ, and YM drafted and revised the manuscript. All authors read and approved the final manuscript.

## Conflict of Interest

The authors declare that the research was conducted in the absence of any commercial or financial relationships that could be construed as a potential conflict of interest.

## Publisher's Note

All claims expressed in this article are solely those of the authors and do not necessarily represent those of their affiliated organizations, or those of the publisher, the editors and the reviewers. Any product that may be evaluated in this article, or claim that may be made by its manufacturer, is not guaranteed or endorsed by the publisher.

## Supplementary Material

Supplementary Table 1 The list of primer sequences used in this study.

Supplementary Figure 1 Expression profiles of the senescence-related genes are detected in various *oss40* mutants by RT-qPCR.

Supplementary Figure 2 Subcellular localization of OsWRKY79 in rice protoplast.

Supplementary Figure 3 Expression profiles of *OsS40* genes are detected in various *oss40* mutants by RT-qPCR.

Supplementary Dataset 1 Expression profiles of the *OsS40* family during rice flag leaf aging.

Supplementary Dataset 2 Expression profiles of the WRKY family in *oss40-14* relative to WT.
