# Supplementary material for: Five OsS40 Family Members Are Identified as Senescence-Related Genes in Rice by Reverse Genetics Approach
Source: Front Plant Sci. 2021 Sep 3;12:701529. doi: 10.3389/fpls.2021.701529 (PMC8446524; doi:10.3389/fpls.2021.701529)
Supplement: Supplementary Table 1 — The list of primer sequences used in this study. [file Data_Sheet_3.pdf]

Supplementary Table S1 The list of primer sequences used in this study

Name and purpose	Forward primer	Reverse Primer
**Primers used for qRT-PCR**		
OsS40-1	ACGACGCTGTGAAGAAGAAGATG	AGCCTCCTCCGATCAACAATG
OsS40-2	GAGGATCAGCCACGGGAGC	TCCCCACGCACACCGAGAA
OsS40-3	CCCAGGGTGAGGTTCCAC	GTCGTCGTATCCGTCGCC
OsS40-4	ACGAGGACGACGAGATGGTG	CAGAGGTCGAGGAATCCGGT
OsS40-5	AACCAATTCCTCGCCGTCC	GTCGTCATCGTCCTCCTCGTC
OsS40-6	TTGTCGCCAACCCAGTCC	CGTCCCACTTGAACATCTCCT
OsS40-7	GCTGTGGCCGGATCACC	GATCTGCTGCTGGCCCG
OsS40-8	GTCGAAGGGCGGGAAGGGGA	CTCCCAGATGGCGTTGCGG
OsS40-9	TAGGCGGTTATCGGTGGAGG	CGAGAAGGTGGTCATCGGTG
OsS40-10	ATGGGCACTTGGGCATTACACC	TCCAGGTCTTGTGCTGCATCTG
OsS40-11	CGACGACGAGGGGAGCAAGA	TCCGAGGGTATCCTCACGGG
OsS40-12	GACGTGCTCTGGCCTGCAT	CGCCTCCGACGGTATCCTC
OsS40-13	CGAGTTCGACGAGTCGGA	CCTGTACTCGACCCCAAGAA
OsS40-14	GTGGAGGAGCTCGACGAGTT	GTGTCTCGTATGGCTGCACC
OsS40-15	GTTCCAGTACCACCACCACC	CGTACCAGACATCAGCCTCC
OsS40-16	TCACGATCAGAGCAACGAAGCG	TCCTCGTGCTGCCTGTTATTCC
Actin	TCCATCTTGGCATCTCTCAG	GTACCCTCATCAGGCATCTG
OSH36	GCACGGAGGCGAACGA	TTGAGCGGTAGCACCCATT
OsNAP	CAAGAAGCCGAACGGTTC	GTTAGAGTGGAGCAGCAT
SGR	AGGGGTGGTACAACAAGCTG	GCTCCTTGCGGAAGATGTAG
NYC1	CATGCAACACCAACAAAAGG	GACCATTCCAGGAGAAGCAG
OsI85	GAGCAACGGCGTGGAGA	GCGGCGGTAGAGGAGATG
OsEIN3	ATCTTCCCGGCAACCTACAA	CATGATCGTGGCATTGTCGT
OsS3H	GCAACGACCGGTATAAGAGC	ATTCGTCGTAGTAGGCCTGG
**Target site Sequences for CRISPR/Cas9**		

OsS40-1	TGGCAGCCGACGATAAGG	GAAGCCCACGACCGAGAC
OsS40-2	TCCTCGTCTCTTCTCCTCCTC	GATCAGGTCTCGAGGAAGCC
OsS40-4	GGGGAGATCAGAGCCAAAGG	CTCGTCGTCCTCGTTGTCG
OsS40-5	CTGGTCAGTAGCCACCACAC	CGACTGGTGGTACGGCTG
OsS40-6	CCCATAAAAGCCAAACCCCC	CTCTTGTCCCCCTCGTGCAA
OsS40-7	GTGCCGGGTCATCCTTCAAT	CAAGGCCCAAACGTCCAAAC
OsS40-8	TCCCCCTGCCGCCGTACTCC	CCGTTGCTTTCGCTTCTCCC
OsS40-10	CTCGATGAAGCCGGTCATGC	ATCTGTTTCCGCTCCTGCTC
OsS40-11	GACCGCCTCCTGCTCTTTC	TCTCAGTCGAGGAATCCGGT
OsS40-12	CGTCCACCCCAACCTCCCCT	CCGCCCACATCACCCCCCTA
OsS40-13	TGCCAGTCCGGTATGTTCAC	GACAACCAAACGTCCAAGGC
OsS40-14	ATGGCGATGGTGGTGG	TCCTTCGATGAACCCG
OsS40-15	CGGCGCTTCTCCTCCTCC	CCGGCTTTCCTCTCCGAC
OsS40-16	CCTCCCCTTGCTCCTCCT	TGTGAGTCTGTTGGTCGGTG
**Primer used for Transcription Activity**		
OsS40-1	CTCGAGGGGGGGCCCATGGAGGAA TTCCAAGAGGC	AGTACCCGGGTACCCATTTCTCGATGA ATCCGGTCA
OsS40-7	CTCGAGGGGGGGCCC ATGGCGACGATGGGAGAG	AGTACCCGGGTACCCA TCCTTCAATGAACCCGGTCA
OsS40-12	CTCGAGGGGGGGCCC ATGGAGCGCCTCCTGCA	AGTACCCGGGTACCCA GTCGAGGAAGCCGGTGC
OsS40-13	CTCGAGGGGGGGCCC ATGGCTGGGAGCGCGA	AGTACCCGGGTACCCA GTCCTCGAAGCCGGTCTTC
OsS40-14	CTCGAGGGGGGGCCC ATGGCGATGGTGGTGG	AGTACCCGGGTACCCA TCCTTCGATGAACCCG
OsS40-15	CTCGAGGGGGGGCCC ATGGCGAAGGCGCGGAA	AGTACCCGGGTACCCA GCCGTCGAAGCCGGTTCG

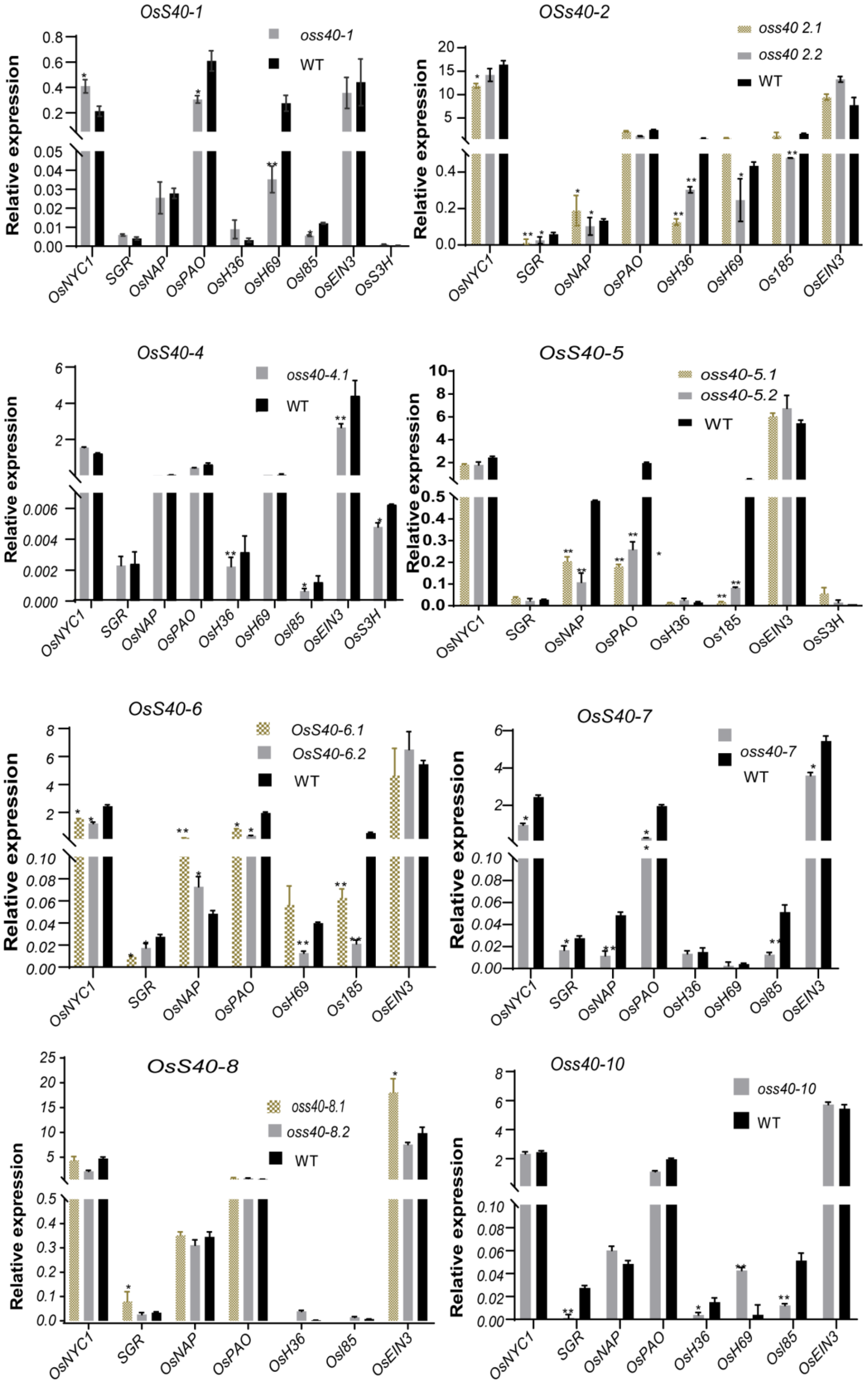

OsS40-1
oss40-1
WT
OSs40-2
oss40 2.1
oss40 2.2
OsS40-4
oss40-4.1
OsS40-5
oss40-5.1
oss40-5.2
OsS40-6
OsS40-6.1
OsS40-6.2
OsS40-7
oss40-7
OsS40-8
oss40-8.1
oss40-8.2
Oss40-10
oss40-10
Relative expression
OsNYC1
SGR
OsNAP
OsPAO
OsH36
OsH69
OsI85
Os185
OsEIN3
OsS3H

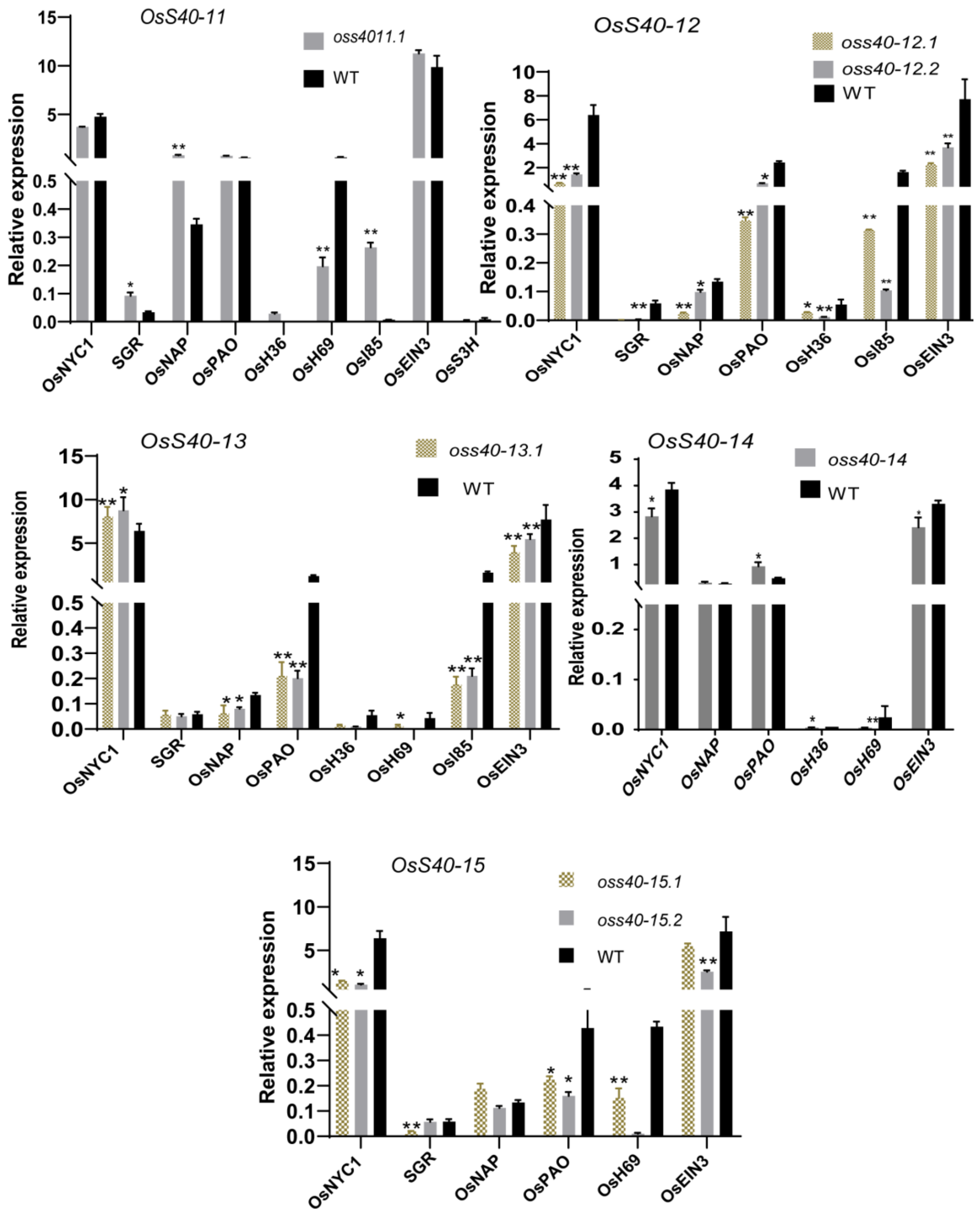

Supplementary Figure S1 Expression profiles of senescence related genes are detected in various oss40s mutants by RT-qPCR. The values are mean ± S.D. Mean and SD values were obtained from three biological replicates and three technical replicates. Significant differences of the expression levels normalized to WT were evaluated using Student's t-test (*, P < 0.05; **, P < 0.01).

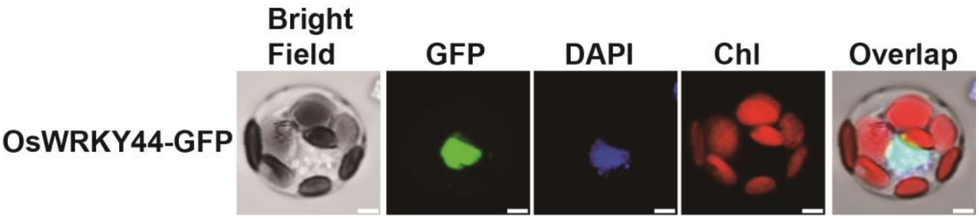

Supplementary Figure S2 Subcellular localization of OsWRKY44 in rice protoplast. Chl, Chlorophyll fluorescence.

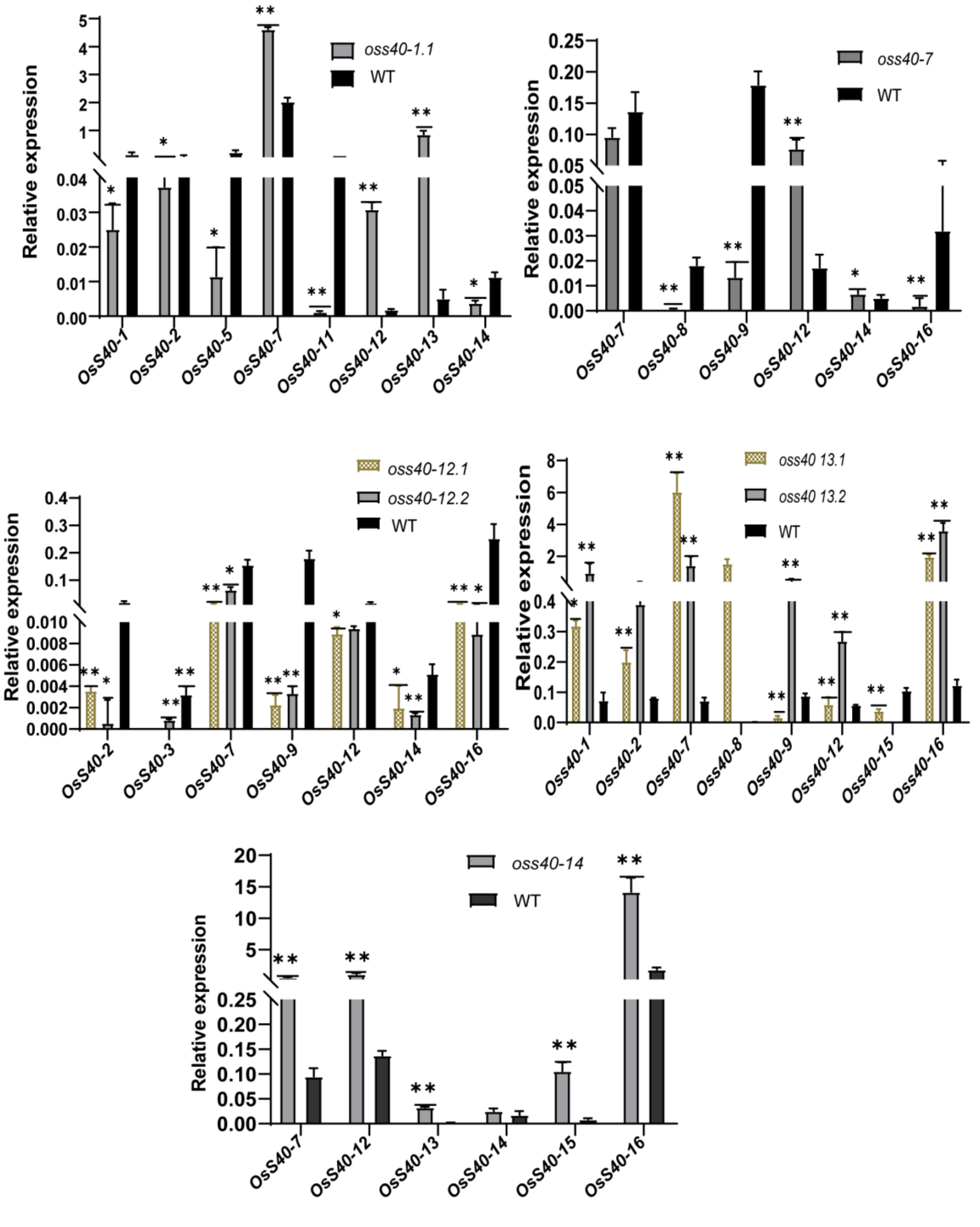

Supplementary Figure S3 Expression profiles of OsS40 genes are detected in various oss40s mutants by RT-qPCR. The values are mean ± S.D. Mean and SD values were obtained from three biological replicates and three technical replicates. Significant differences of the expression levels normalized to WT were evaluated using Student's t-test (*, $P < 0.05$; **, $P < 0.01$).
